# Supplementary material for: Current clinical practice in managing somatosensory impairments and the use of technology in stroke rehabilitation
Source: PLoS One. 2022 Aug 11;17(8):e0270693. doi: 10.1371/journal.pone.0270693 (PMC9371309; doi:10.1371/journal.pone.0270693)
Supplement: S1 File — (PDF) [file pone.0270693.s001.pdf]

Survey Questionnaire: Robotic-based Exercises for Proprioceptive Retraining Post Stroke

Thank you for helping us out in completing the questionnaire for our research study.

Although all questions are optional, please try your best to answer as many as possible to help us with data collection for this study.

The objectives of this study are (1) to understand standard clinical practices in managing impairments in somatosensation; and (2) to explore if therapists have used recent technology in their practice as both an intervention, and as a means to objectively assess patient.

The questionnaire is divided into 4 parts. It will take about 10 – 15 minutes to complete. All answers will be kept confidential and used only for research purpose.

At the end of the questionnaire, you will be asked to provide a pseudonym to claim your $10 voucher.

Participant Information Sheet and Consent Form

1. You are invited to participate in a research study on therapists use of robotic technology in stroke rehabilitation. This study is conducted by Ananda Sidarta, PhD, from Rehabilitation Research Institute of Singapore, Nanyang Technological University.

   This study will take approximately 15 minutes of your time. You will be asked to complete an online survey about the type of somatosensory retraining provided to stroke patients, the perceived usefulness of proprioceptive training post-stroke, and the use of robotic technology in stroke rehabilitation, particularly in proprioceptive training. Occupational therapists and physiotherapists with at least 1 year of experience, currently working with stroke patients in Singapore are invited to take part.

   Your participation in this study is completely voluntary, and you have the right to withdraw from the study at any time without any penalty. You may skip any questions you do not wish to answer. If you do not wish to complete this survey, just close your browser.

   Your participation in this research will be kept confidential, and data will be averaged and reported in aggregate. Your responses will be anonymous and IP addresses will not be collected to guarantee complete anonymity. Possible outlets of dissemination may be in local scientific conferences and/or as a journal manuscript. Although your participation in this research may not benefit you personally, it will help us understand the use of robotic technology in somatosensory/ proprioceptive training post-stroke as a means of objectively quantifying the assessment of patients' performance, and as an intervention for improving patients' somatosensory/ proprioceptive performance. You will be compensated $10 for the time and effort taken to participate in the study.

   There are no known risks to individuals participating in this survey beyond those existed in daily life.

   If you have questions about this project, you may contact the principal investigator, Ananda Sidarta, PhD at ananda.sidarta@ntu.edu.sg or 6904 1361.

   This project has been reviewed and approved by NTU-Institutional Review Board. Questions concerning your rights as a participant in this research may be directed to the NTU-IRB at IRB@ntu.edu.sg or call 6592 2495.

   By selecting agree, you certify that you are 21 years old or older and have read and understood the above, and it indicates your willingness to voluntarily take part in the study.

   ☐ Agree

Part 1 – Demographics

2. Please indicate your gender.

☐ Female ☐ Male

3. Please indicate your age.

Click or tap here to enter text.

4. What is your profession?

☐ Occupational therapist ☐ Physiotherapist

5. How many years of experience do you have working with stroke patients in Singapore?

Click or tap here to enter text.

6. Please indicate your current practice setting.

☐ Acute / Restructured Hospital
☐ Community Hospital
☐ Day Rehabilitation Centre
☐ Nursing Home
☐ Other: Click or tap here to enter text.

7. What type of stroke patients do you currently work with?

☐ Acute
☐ Subacute
☐ Chronic

8. What is your current percentage of clinical service time in general with stroke patients?

☐ < 25%
☐ 25 – 50%
☐ 51 – 75%
☐ > 75%

Part 2 – Somatosensory Impairments

Note: This part focuses on clinical practices that aim at improving functions of bodily sensations (*somatosensation*) such as tactile, proprioception and kinaesthesia, object recognition, etc.

9. What type of somatosensory retraining have you applied to stroke patients in the clinic? Check all that apply.

☐ **Tactile-based exercises, or discrimination.** E.g., discriminating textures or vibration, localization of touch or pressure, exercise focusing on tactile extinction.

☐ **Proprioception and kinaesthesia.** E.g., joint position sense discrimination, recognizing direction of limb movements.

☐ **Object discrimination, or recognition.** E.g., active object exploration by touch, recognizing shape, size, and weight.

☐ **Use of compression.** E.g., pneumatic, compressive garment or devices on the affected limb.

☐ **Thermal-based intervention** (sensation of hot and cold).

☐ **Electrical/magnetic stimulation.** E.g., transcutaneous electrical nerve stimulation (TENS), repetitive peripheral magnetic stimulation (RPMS).

☐ **Balance-related training**.

☐ **Movement-based exercises** that may include, E.g., repetitive active movements of shoulder flexion/extension, knee flexion/extension.

☐ **Functional, task-specific training.** E.g., holding a knife and fork, uni-manual or bi-manual tasks, walking or gait.

☐ Other: Click or tap here to enter text.

10. If somatosensory retraining is part of the required intervention for stroke patients who need it, what is the average proportion of time you spend per week?

☐ < 25%
☐ 26 – 50 %
☐ 51 – 75 %
☐ > 75 %
☐ Unsure

11. If you checked 'unsure' on the previous question, please elaborate:

Click or tap here to enter text.

12. Please indicate how frequently you deliver the following somatosensory-related intervention to your stroke patients.

	Not available	Rarely <2x/week	Sometimes 2-5x/week	Regularly > 5x/week
Tactile based	☐	☐	☐	☐
Proprioception	☐	☐	☐	☐
Functional tasks	☐	☐	☐	☐
Electrical/magnetic stimulation	☐	☐	☐	☐

13. What type of standard somatosensory assessment do you usually conduct in the clinic? Check all that apply.

☐ Semmes-Weinstein Monofilament (SWM) Test
☐ Nottingham Sensory Assessment, including original or revised version
☐ Rivermead Assessment of Somatosensory Performance
☐ Fugl-Meyer Assessment for Sensation (UL or LL)
☐ Non-standardized tests
☐ Other: Click or tap here to enter text.

14. If you usually perform non-standardized assessments, please indicate below:

☐ Light touch
☐ Pressure
☐ Pain
☐ Position sense
☐ Stereognosis
☐ Sensory extinction
☐ Other: Click or tap here to enter text.

Part 3 – Retraining of Limb Position Senses

The following section focuses on **position senses** (proprioception, kinaesthesia).

15. Please indicate your level of agreement with the following statements based on its perceived importance to you.

	Strongly disagree	Disagree	Neutral	Agree	Strongly agree
Intervention in proprioception can yield improvement in motor functions after a stroke.	☐	☐	☐	☐	☐
Intervention in proprioception is essential for patients with sensory impairment.	☐	☐	☐	☐	☐
An effective and highly repetitive proprioceptive *intervention* is beneficial clinically.	☐	☐	☐	☐	☐
An objective and reliable proprioceptive *assessment* is beneficial clinically.	☐	☐	☐	☐	☐
It is important to track and monitor improvements in proprioception *over time*.	☐	☐	☐	☐	☐

Part 4 – Use of Technology in Rehabilitation

The following section focuses on the use of recent technologies in the rehabilitation setting. This includes, but is not limited to, game interfaces and robotic devices that aid therapy in the clinics.

16. How long have you been using rehabilitation technologies in your clinical work?

☐ Never ☐ < 1 year ☐ 1 – 3 years ☐ > 3 years

17. Do you use technology as **intervention** (either main or supplement) on any of the following areas with stroke patients? Check all that apply.

☐ Cognition
☐ Upper extremity movement (e.g., reaching, grasping)
☐ Sensation (e.g., tactile, proprioception)
☐ Lower extremity movement (e.g., gait, mobility)
☐ Balance
☐ Functional activities (e.g., manipulating objects, opening jars, etc.)
☐ I never use technology as intervention

18. Do you use technology as an **assessment** tool (either main or supplement) on any of the following areas with stroke patients? Check all that apply.

☐ Cognition
☐ Upper extremity movement (e.g., reaching, grasping)
☐ Sensation (e.g., tactile, proprioception)
☐ Lower extremity movement (e.g., gait, mobility)
☐ Balance
☐ Functional activities (e.g., manipulating objects, opening jars, etc.)
☐ I never use technology as an assessment tool

19. What forms of rehabilitation technology do you use clinically?

	Not Available	Available but not used	Rarely < 2x/week	Sometimes 2-5x/week	Regularly > 5x/week
Upper limb robotics (e.g., Armeo-Spring)	☐	☐	☐	☐	☐
Lower limb/ gait rehab robotics (e.g., Lokomat)	☐	☐	☐	☐	☐
Virtual reality games and commercial system (e.g., Wii or Kinect)	☐	☐	☐	☐	☐
Electrical stimulation (e.g., NMES, TENS)	☐	☐	☐	☐	☐

20. Is there any other form of rehabilitation technology you use that has not been covered above? (Please elaborate on one type of technology and frequency of its usage.)

    Click or tap here to enter text.

21. Please indicate your level of agreement with the following statements which relate to your perspectives/ thoughts on the use of **robotic technology** in stroke rehabilitation.

	Strongly disagree	Disagree	Neutral	Agree	Strongly agree
Robotic-based therapy can provide effective and highly repetitive *intervention*.	☐	☐	☐	☐	☐
Robotic technology can be used as an objective and reliable *assessment* tool.	☐	☐	☐	☐	☐
Robotic-based therapy *combined* with conventional therapy can be more effective than conventional therapy alone.	☐	☐	☐	☐	☐
Robotic-based therapy can keep patients motivated and engaged.	☐	☐	☐	☐	☐

22. Please indicate your level of agreement with the following statements which relate to your perspectives/ thoughts on the use of robotic technology <u>specifically</u> for proprioception.

	Strongly disagree	Disagree	Neutral	Agree	Strongly agree
Robotic technology would be beneficial in retraining of proprioception.	☐	☐	☐	☐	☐
An integrated motor and sensory rehabilitation program would be beneficial for patients.	☐	☐	☐	☐	☐

23. If any, what would you think the perceived main barriers to incorporating robotic-based rehabilitation in the clinic? Check all that apply.

☐ Price
☐ Space
☐ Usability (ease of use)
☐ Patient's needs
☐ Inadequate technical support (e.g., training etc.)

24. Comments (**<u>if any</u>**) on somatosensory retraining and the use of technology in stroke rehabilitation.

Click or tap here to enter text.

Participant Pseudonym

In this last section, you will be asked to create a pseudonym for voucher claim purpose.

25. Please create a pseudonym using 3 random digits, followed by "ACTSENS", and 01 (if you currently work in inpatient setting) or 02 (if you currently work in outpatient setting). For example, 789ACTSENS02. Remember to note down your pseudonym before submitting your response.

    Note: You will be notified of the date and time to collect your voucher. Unfortunately, you cannot redeem the voucher if you forget the pseudonym.

    Click or tap here to enter text.

Thank you for your time, your response has been submitted.
