# Supplementary material for: Current clinical practice in managing somatosensory impairments and the use of technology in stroke rehabilitation
Source: PLoS One. 2022 Aug 11;17(8):e0270693. doi: 10.1371/journal.pone.0270693 (PMC9371309; doi:10.1371/journal.pone.0270693)
Supplement: S4 File — (DOCX) [file pone.0270693.s004.docx]

This supporting information file contains several figures and tables generated from the additional comparative analyses. Occupational therapists and physiotherapists’ views and practices on somatosensory assessment and intervention, and the adoption of technology in different regional healthcare clusters were further analysed and indicated below. The regional healthcare clusters are indicated as ‘Central’, ‘West’, ‘East’, and ‘Nationwide’ (multiple, distributed across different regions).


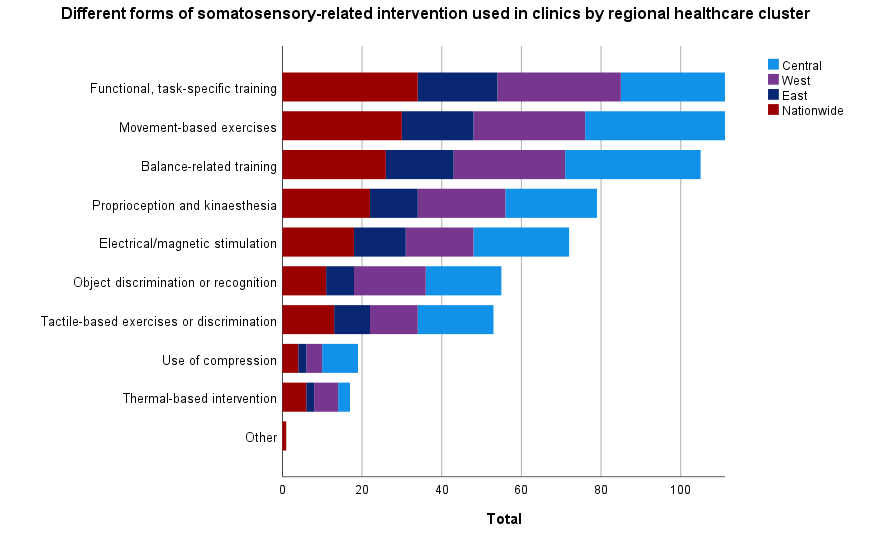


**Fig A. Different forms of somatosensory-related intervention used in clinics by regional healthcare cluster.** The number of responses did not always sum to 132 as participants were allowed to select multiple answers.


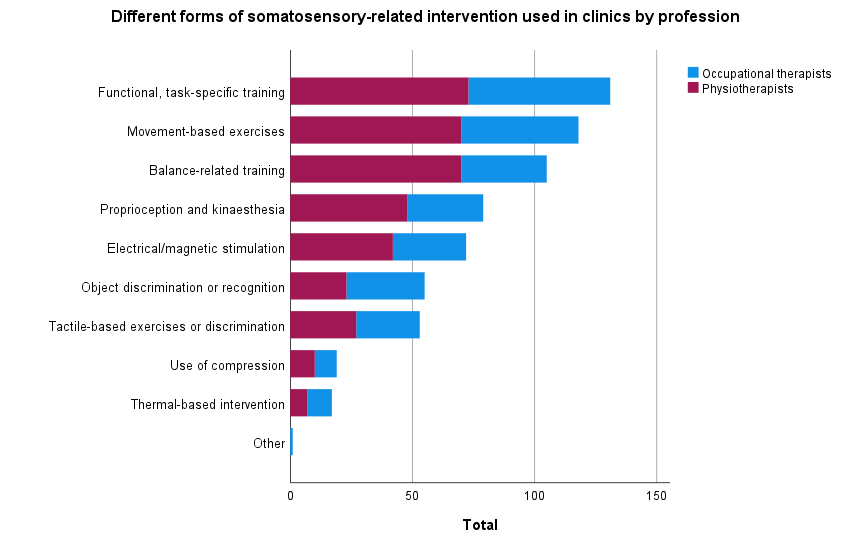


**Fig B. Different forms of somatosensory-related intervention used in clinics by profession.** The number of responses did not always sum to 132 as participants were allowed to select multiple answers.

**Table A.** **Types of standardised and non-standardised assessment of somatosensation by regional healthcare cluster.**

|  | **Central** | | | **West** | | **East** | | **Nationwide** | |
| --- | --- | --- | --- | --- | --- | --- | --- | --- | --- |
|  | ***n*** | **%** | ***n*** | | **%** | ***n*** | **%** | ***n*** | **%** |
| Standardised |  |  |  | |  |  |  |  |  |
| Fugl-Meyer Assessment for Sensation | 42 | 91.3 | 28 | | 90.3 | 18 | 90.0 | 30 | 85.7 |
| Nottingham Sensory Assessment | 34 | 73.9 | 28 | | 90.3 | 17 | 85.0 | 26 | 74.3 |
| Rivermead Assessment of Somatosensory Performance | 23 | 50.0 | 22 | | 71.0 | 12 | 60.0 | 22 | 62.9 |
| Semmes-Weinstein Monofilament Test | 24 | 52.2 | 17 | | 54.8 | 13 | 65.0 | 18 | 51.4 |
| Non-standardised tests | 46 | 100.0 | 31 | | 100.0 | 20 | 100.0 | 34 | 97.1 |
| Non-standardised |  |  |  | |  |  |  |  |  |
| Light touch | 26 | 56.5 | 27 | | 87.1 | 11 | 55.0 | 30 | 85.7 |
| Position sense | 23 | 50.0 | 26 | | 83.9 | 5 | 25.0 | 32 | 91.4 |
| Pain | 11 | 23.9 | 19 | | 61.3 | 0 | 0.0 | 21 | 60.0 |
| Pressure | 8 | 17.4 | 18 | | 58.1 | 1 | 5.0 | 18 | 51.4 |
| Sensory extinction | 15 | 32.6 | 13 | | 41.9 | 1 | 5.0 | 14 | 40.0 |
| Stereognosis | 7 | 15.2 | 12 | | 38.7 | 2 | 10.0 | 13 | 37.1 |
| Other | 0 | 0.0 | 0 | | 0.0 | 1 | 5.0 | 2 | 5.7 |

*Note.* Participants were allowed to select multiple answers.

**Table B.** **Types of standardised and non-standardised assessment of somatosensation by profession.**

|  | **Occupational therapists** | | **Physiotherapists** | |
| --- | --- | --- | --- | --- |
|  | ***n*** | **%** | ***n*** | **%** |
| Standardised |  |  |  |  |
| Fugl-Meyer Assessment for Sensation | 29 | 50.0 | 14 | 18.9 |
| Nottingham Sensory Assessment | 5 | 8.6 | 2 | 2.7 |
| Rivermead Assessment of Somatosensory Performance | 2 | 3.4 | 3 | 4.1 |
| Semmes-Weinstein Monofilament Test | 3 | 5.2 | 0 | 0.0 |
| Non-standardised tests | 57 | 98.3 | 72 | 97.3 |
| Non-standardised |  |  |  |  |
| Light touch | 41 | 70.7 | 53 | 71.6 |
| Pressure | 23 | 39.7 | 22 | 29.7 |
| Pain | 23 | 39.7 | 28 | 37.8 |
| Position sense | 34 | 58.6 | 52 | 70.3 |
| Stereognosis | 20 | 34.5 | 14 | 18.9 |
| Sensory extinction | 14 | 24.1 | 29 | 39.2 |
| Other | 1 | 1.7 | 2 | 2.7 |

*Note.* Participants were allowed to select multiple answers.


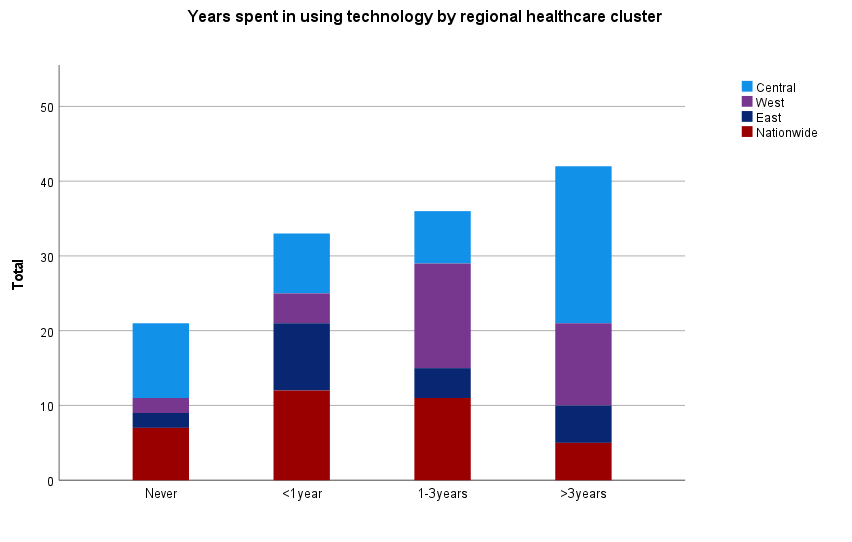


**Fig C. Years spent in using technology by regional healthcare cluster.** The number of responses did not always sum to 132 as participants were allowed to select multiple answers.


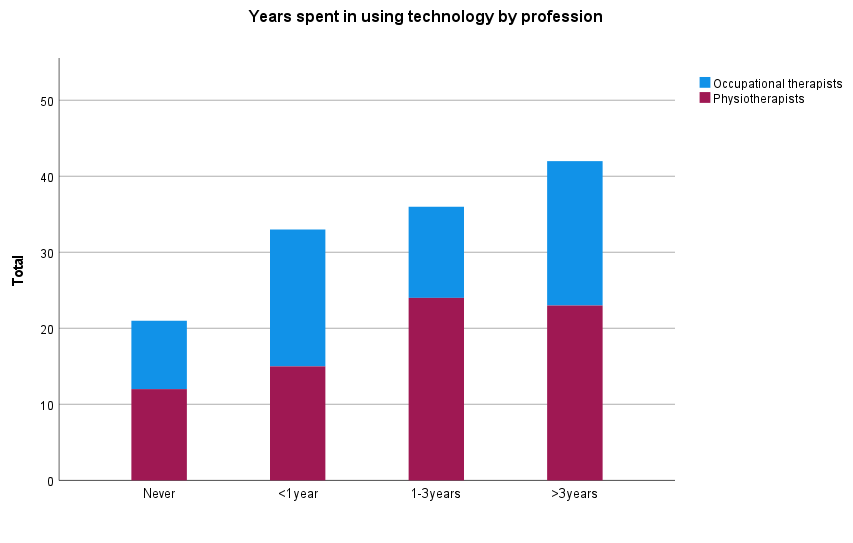


**Fig D. Years spent in using technology by profession.** The number of responses did not always sum to 132 as participants were allowed to select multiple answers.


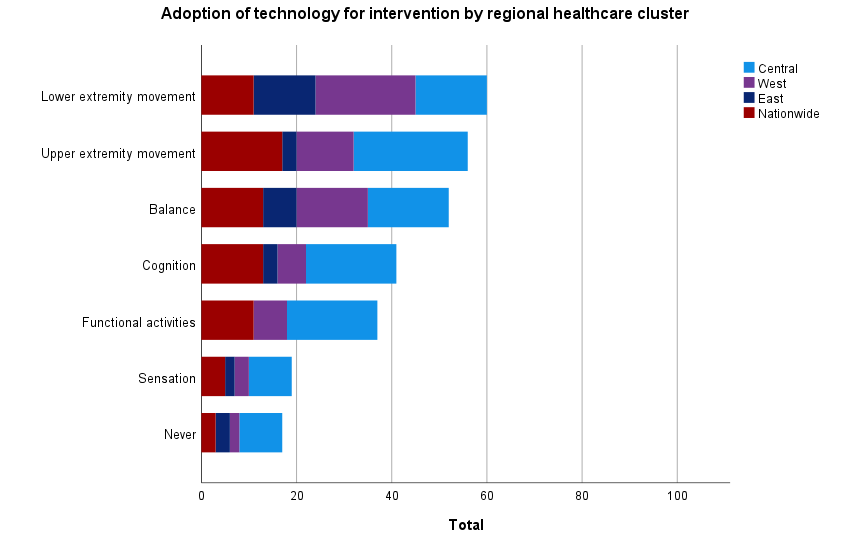


**Fig E. Adoption of technology for intervention by regional healthcare cluster.** The number of responses did not always sum to 132 as participants were allowed to select multiple answers.


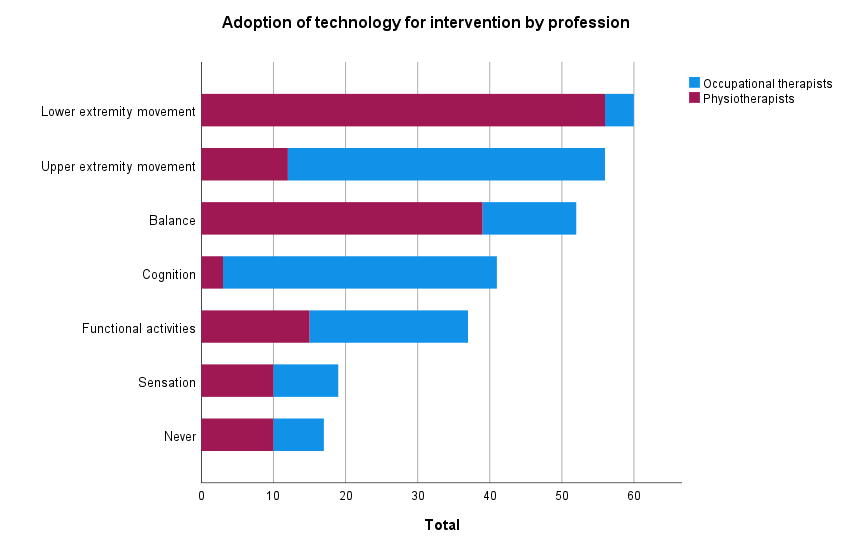


**Fig F. Adoption of technology for intervention by profession.** The number of responses did not always sum to 132 as participants were allowed to select multiple answers.


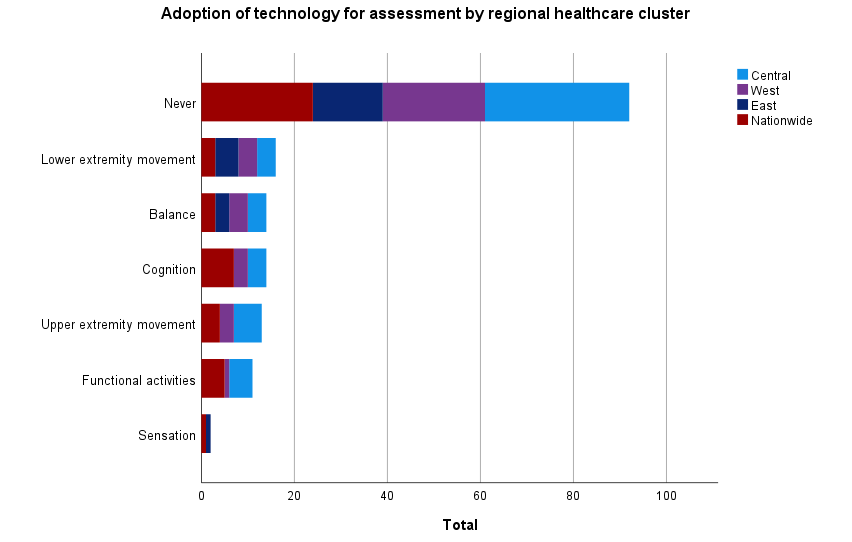


**Fig G. Adoption of technology for assessment by regional healthcare cluster.** The number of responses did not always sum to 132 as participants were allowed to select multiple answers.


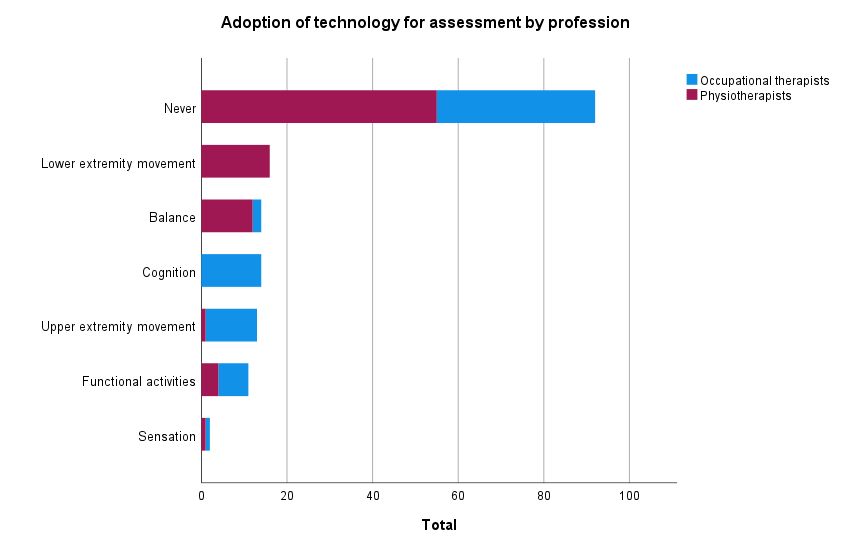


**Fig H. Adoption of technology for assessment by profession.** The number of responses did not always sum to 132 as participants were allowed to select multiple answers.


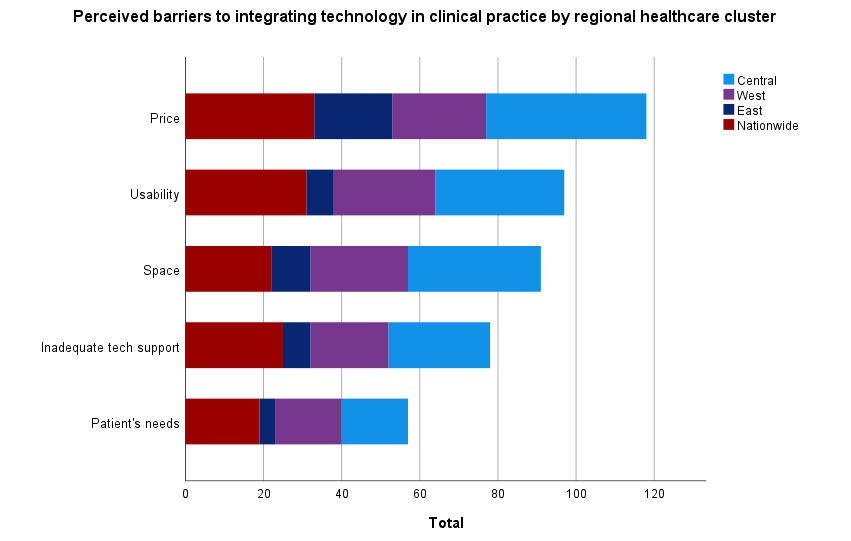


**Fig I. Perceived barriers to integrating technology in clinical practice by regional healthcare cluster.** The number of responses did not always sum to 132 as participants were allowed to select multiple answers.


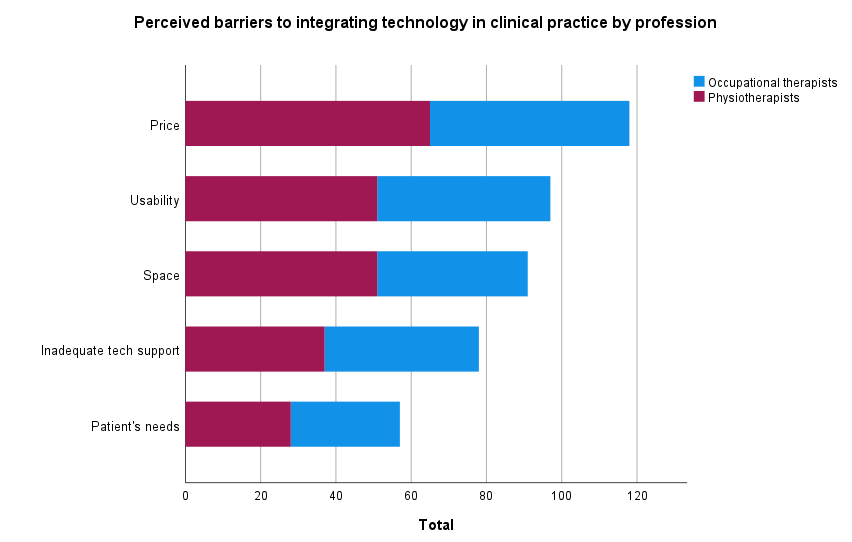


**Fig J. Perceived barriers to integrating technology in clinical practice by profession.** The number of responses did not always sum to 132 as participants were allowed to select multiple answers.
